# Pulmonary functions in patients with diabetes mellitus

**DOI:** 10.4103/0970-2113.80314

**Published:** 2011

**Authors:** Muhammad Irfan, Abdul Jabbar, Ahmed Suleman Haque, Safia Awan, Syed Fayyaz Hussain

**Affiliations:** *Department of Medicine, The Aga Khan University Hospital, Karachi, Pakistan*; 1 *Department of Medicine, Kettering General Hospital and University of Leicester, United Kingdom*

**Keywords:** Diabetes mellitus, pulmonary function test, spirometry, vital capacity

## Abstract

**Background:**

A reduction in lung capacity has been reported previously among diabetics. According to WHO estimates, Pakistan is currently eighth in the prevalence of diabetes mellitus (DM) and will become fourth by the year 2025 with over 15 million individuals. This study was designed to see the impairment of lung functions on spirometry in DM patients.

**Objective:**

Our aim was to investigate the pulmonary functions tests of Pakistani patients with DM.

**Materials and Methods:**

Between January to July 2004, 128 subjects who were never-smokers and had no acute or chronic pulmonary disease were recruited. Sixty-four of these subjects had DM and 64 were healthy matched controls. All underwent screening with detailed history, anthropometry, lipid profile, and spirometric measurements at the Aga Khan University Hospital, Karachi, Pakistan.

**Results:**

The mean age of diabetics and matched control were 54.3±9 and 54.0±8 (*P*<0.87) years, respectively. Diabetes patients showed a significant reduction in the forced vital capacity (FVC) [mean difference (95% CI) – 0.36 (–0.64, –0.07) *P*<0.01], forced expiratory volume in one second (FEV_1_) [– 0.25(–0.50, –0.003) *P*<0.04], and slow vital capacity (SVC) [– 0.28(–0.54, –0.01) *P*<0.04], relative to nondiabetic controls. There was no significant difference noted in the forced expiratory ratio and maximum mid-expiratory flow between the groups. There was also a significant higher level of triglycerides noted among diabetics (*P*<0.001).

**Conclusion:**

Diabetic patients showed impaired lung function independent of smoking. This reduced lung function is likely to be a chronic complication of diabetes mellitus.

## INTRODUCTION

The association of reduced lung function and diabetes mellitus (DM) has been described for many years.[1] Several studies have suggested that diabetes is associated with impaired pulmonary function.[2–10] Davis *et al*.[2] suggested that the lung is a target organ in DM and that glycemic exposure is a strong determinant of reduced pulmonary function in type 2 patients. Theoretically, several pathological changes may affect the lungs in patients with DM. Ljubic *et al*.[11] showed that diabetes could lead to the development of pulmonary complications due to collagen and elastin changes. Another theory suggested that increased nonenzymatic glycation of proteins and peptides of the extracellular matrix at chronic high circulating glucose levels may also have an important role in the pathological changes of the lungs in DM patients.[12]

The prevalence of DM in Pakistan is reported as high as 10% among adults with an equal number of people suffering from glucose intolerance.[13,14] According to WHO estimates, Pakistan is currently eighth in the prevalence of DM and will become fourth by the year 2025 with over 15 million individuals.[15] Since the prevalence of diabetes in Pakistan is high, it would be important to study pulmonary functions in this population. The present study was, therefore, undertaken to investigate pulmonary functions in Pakistani patients with DM.

## MATERIALS AND METHODS

This study was conducted in the Department of Medicine, Aga Khan University Hospital (AKUH), Karachi, Pakistan, during January to July 2004. One hundred twenty-eight subjects (>30 years) who were never-smokers and had no acute or chronic pulmonary disease were recruited. Sixty-four of these subjects had DM and 64 were healthy matched controls. The patients with DM were selected from the outpatient department of AKUH. Healthy matched controls were selected from the Executive Clinic of AKUH. They were non-smokers, non–diabetics, and had no history of any acute or chronic lung disease and they came for their routine check ups.

The detailed history and physical examination was carried out by a specialist physician. All patients having any acute or chronic pulmonary disease, and smokers (defined as smoking of any number of cigarettes) were excluded. Informed consent was taken from all subjects.

The study was approved by Institutional Review Board (IRB) of Aga khan University.

### Anthropometric measurements and biochemical profile

Standing height and weight of the patient were measured and body mass index (BMI) was calculated. Fasting and random blood sugars and fasting lipid profile were checked.

### Pulmonary function test

Pulmonary functions including forced vital capacity (FVC), forced expired volume in one second (FEV_1_), FEV_1_/FVC ratio, slow vital capacity (SVC), and peak expiratory flow rate (PEFR) were measured by spirometer MedGraphics Profiler (pulmonary diagnostic system by Medical Graphic Corporation, USA) according to the American Thoracic Society (ATS) criteria.[16]

Spirometry was performed before and 15 min after inhalation of 0.2 mg salbutamol inhaler (Made by GlaxoSmithKline, Pakistan) at room temperature ranged from 19°C to 24°C, with a mean of 22±0.5. The subject breathed in from room air and then exhaled into the spirometer. The wedge opened as air was blown into the spirometer, and a marker moved accordingly along a sheet of paper for 6 seconds. The spirometer was computerized and printed the FEV_1_ and FVC values after the forced expiration had been performed. There was no time lag between the onset of forced expiration and the onset of timing for FEV_1_. No extrapolation was performed. Best of three satisfactory readings was taken for the analysis. Highest value for FVC and the highest value for FEV_1_ were used in the ratio FEV_1_/ FVC. The variables were reported in absolute volume as well as the percent predicted based on the regression equations.

### Statistical analysis

Statistical analysis was performed using SPSS version 15.0 for windows (SPSS Inc., Chicago, IL, USA). Patients with diabetes were compared with those without diabetes for all subjects using independent *t*-test. For the analysis, γ^2^-test and Fisher’s exact test were used for categorical variables. Logistic regression was used to examine the patients with diabetes with those without diabetes.

## RESULTS

Sixty four patients with DM (37 male) and 64 matched controls (39 male) were selected. The mean age of diabetic patients and matched control were 54.3±9 and 54.0±8 (*P*<0.87) years, respectively. Demographics and biochemical profile of both groups are presented in Table 1. Diabetic patients have higher blood pressures (*P*<0.001) than nondiabetics. There was also a significant higher level of triglycerides noted among diabetics (*P*<0.001).

**Table 1:** Demographics and biochemical profile of diabetics and nondiabetics

Variables	DM nonsmoker Mean±SD	Non DM nonsmoker Mean±SD	Mean difference (95% CI)	*P* value
Gender				
Male	37 (57.8)	39 (60.9)		0.71
Female	27 (42.2)	25 (39.1)		
Age, years	54.31±9.98	54.058.37	0.26 (-2.95, -3.49)	0.87
BMI	29.06±6.05	28.20±4.23	0.85 (-0.971, -2.688)	0.35
Systolic BP	138.8±17.78	121.95±14.41	16.85 (10.58, -23.12)	<0.001
Diastolic BP	82.34±9.68	78.27±10.03	4.07 (0.14, -8.00)	0.04
FBS	167.09±55.4	102.55±9.51	64.54 (49.18, -79.89)	<0.001
RBS	214.04±89.56	108.4±21.78	105.64 (80.41, -130.88)	<0.001
LDL	112.11±25.77	122.92±28	-10.81 (-21.75, -0.128)	0.05
TG	196.05±102.03	133±51	63.04 (28.84, -97.24)	<0.001
Cholestrol	181.86±29.8	193.66±33.3	-11.79 (-24.65, -1.06)	0.07

BMI: Body mass index; BP: Blood pressure; FBS: Fasting blood sugar, random blood sugar; LDL: Low-density lipoprotein; TG: Triglyceride. Figures in parenthesis are in percentage

On spirometry, diabetic patients showed a significant reduction in the forced vital capacity (FVC) [mean difference (95% CI) – 0.36 (–0.64, –0.07) *P*<0.01], forced expiratory volume in one second (FEV_1_) [mean difference (95% CI) – 0.25 (–0.50, –0.003) *P*<0.04], and slow vital capacity (SVC) [mean difference (95% CI) – 0.28 (–0.54, –0.01) *P*<0.04], relative to nondiabetic controls. There was no significant difference noted in the forced expiratory ratio (FEV1/FVC) and maximum mid expiratory flow (MMEF) between the groups [Table 2].

**Table 2:** Pulmonary functions test in diabetics and nondiabetics

Variables	DM nonsmoker Mean±SD	Non DM non-smoker Mean±SD	Mean difference (95% CI)	*P* value
FVC	2.46±0.83	2.82±0.80	-0.36 (-0.64, -0.07)	0.01
FEV_1_	2.04±0.75	2.29±0.67	-0.25 (-0.50, -0.003)	0.04
FEV_1_/FVC	81.94±8.26	81.40±7.16	0.54 (-2.16, -3.25)	0.69
SVC	2.60±0.76	2.88±0.77	-0.280 (-0.54, -0.012)	0.04
MMEF	2.49±1.30	2.48±0.97	0.001 (-0.40, -0.406)	0.99

FVC: Forced vital capacity; FEV_1_: Forced expiratory volume in 1 second; SVC I: Slow vital capacity; MMEF: Maximum mid-expiratory flow

## DISCUSSION

Our study shows decreased pulmonary function impairment in diabetic patients. This association is explained by age, sex, and BMI. Diabetics showed a significant reduction in FVC, FEV1, and SVC, relative to their matched controls. However FEV_1_/FVC was less in diabetics but was statistically nonsignificant.

Our results confirm the results observed in other studies that showed decreased pulmonary function in diabetics.[2–10] Pakistani diabetic patients behave in a similar way as reported in other communities.

Meo *et al*.[3,4] in their studies on Saudi diabetic patients showed significant reduction in FVC, FEV1, and PEF, as compared to their matched controls. They also showed a strong association with a dose–effect response of duration of disease and decreased pulmonary function impairment in their diabetic patients. A study published from the neighboring country India[17] showed a significant reduction in DLco in diabetic patients as compared to the controls but they failed to show any differences among the groups for other pulmonary functions; FVC, FEV1, PEF, and maximal static inspiratory and expiratory pressures. The major limitation in that study was a very small number of patients in each group.

Davis *et al*.[2] conducted a large community-based study in Western Australia in type 2 diabetic patients and demonstrated that VC, FVC, FEV1, and PEF were decreased in type 2 diabetic patients. They also suggested that the reduced lung volumes and airflow limitation are likely to be chronic complications of type 2 diabetes.

Asanuma[9] also reported that FVC and FEV1 were reduced in Japanese diabetic subjects compared to control subjects.

The pathophysiology for reduced lung functions in diabetics is still not very clear but there have been some reports of histopathological changes in the lungs of diabetic patients, including basal lamina thickening[18] and fibrosis.[19] Impairment in lung function of patients with diabetes are believed to be the consequence of biochemical alterations in the connective tissue constituents of the lung, particularly collagen and elastin, as well as microangiopathy due to the nonenzymatic glycosylation of proteins induced by chronic hyperglycemia.[6,11,20,21] The functional abnormalities ensuing from these changes manifest clinically by way of a reduction in elastic recoil of the lung, lung volumes, and pulmonary capacity for the diffusion of carbon monoxide.[21] The concomitant pulmonary structural impact of these biochemical alterations, described to date, consist of a thickening of the alveolar epithelial basal lamina[22] and a specific type of nodular fibrosis of the lung.[19] Autonomic and phrenic neuropathy causing alterations in bronchial reactivity and respiratory muscle function was also suggested in one study.[23]

In our study, diabetic patients had significant hypertriglyceridemia as compared to controls. This finding was not observed in other studies. Sinha *et al*.[17] in their study showed a significant correlation between decreased DLco and cholesterol level. Dyslipidemia might have a contributory role in the pathogenesis of decreased lung functions in diabetic patients.

Our study has several limitations. First, there are less number of patients so we cannot generalize the result in different ethnic groups of Pakistan. Second, we have not checked the DLco in our patients. Several studies showed that DLco is significantly reduced in diabetic patients, even in patients with normal spirometric values. DLco was not done because the cost issues may not be available in most developing country settings. Third, we have not checked the association between the glycemic control and reduced lung function.

In summary, our study also supports the other studies that diabetic patients showed impaired lung function independent of smoking. There was a decrease in FVC, FEV_1_, and SVC as compared to their controls. This reduced lung function is likely to be a chronic complication of diabetes mellitus. Lung functions need to be checked periodically to assess the severity of impairment. There is a need of larger prospective study with long observational course to confirm these observations.
